# Significant response to nivolumab for metastatic chromophobe renal cell carcinoma with sarcomatoid differentiation: a case report

**DOI:** 10.1186/s12894-018-0339-2

**Published:** 2018-04-18

**Authors:** Go Noguchi, Sohgo Tsutsumi, Masato Yasui, Shinji Ohtake, Susumu Umemoto, Noboru Nakaigawa, Masahiro Yao, Takeshi Kishida

**Affiliations:** 1 Department of Urology, Kanagawa Cancer Center, 2-3-2, Nakao, Asahi-ku, Yokohama, Kanagawa 2418515 Japan; 2 Department of Urology, Yokohama City University Graduate School of Medicine, Yokohama, Japan

**Keywords:** Non-clear renal cell carcinoma, Sarcomatoid differentiation, Immune-checkpoint inhibitor, Nivolumab

## Abstract

**Background:**

The treatment of advanced or metastatic renal cell carcinoma (RCC) has drastically changed since the approval of immune checkpoint therapy. Nivolumab is a treatment option for patients with metastatic RCC, previously treated with targeted antiangiogenic therapy. The efficacy of nivolumab for patients with RCC was established by the Checkmate 025 clinical trial. Chromophobe RCC (CRCC) represents around 5% of RCC cases, but non-clear cell RCC (non-ccRCC) subtypes were excluded from the Checkmate 025 clinical trial. We report a case in which the use of nivolumab as the seventh-line therapy elicited a significant response in the treatment of metastatic CRCC with sarcomatoid differentiation.

**Case presentation:**

We report a case of a 41-year-old woman with metastatic CRCC with sarcomatoid differentiation. She was treated with sunitinib, pazopanib, everolimus, sorafenib, axtinib, and temsirolimus, but treatment was discontinued because of disease progression or strong adverse events. Seventh-line treatment with nivolumab was initiated and significant clinical improvement was noted after 4 cycles. The treatment was well-tolerated with no significant side effects and the patient continues with nivolumab treatment at present.

**Conclusions:**

Nivolumab may be an attractive treatment option for non-ccRCC patients with sarcomatoid differentiation that exhibited aggressive characteristics and poor prognosis. Further investigation is warranted.

## Background

The treatment of advanced or metastatic renal cell carcinoma (RCC) has been drastically changed by the approval of immune checkpoint therapy. Nivolumab, the fully humanized monoclonal immunoglobulin(Ig)-G4 programmed death 1 (PD-1) checkpoint inhibitor, is a treatment option for patients with metastatic RCC previously treated with targeted antiangiogenic therapy. The efficiency of nivolumab for patients with RCC was established by the Checkmate 025 clinical trial [1]. Chromophobe RCC (CRCC) represents a heterogeneous RCC subtype and comprises about 5% of cases of RCC, but non-clear cell subtypes including CRCC were excluded from the Checkmate 025 trial [1]. To date, only one case of CRCC successfully treated with nivolumab has been reported [2]. We present a case of a patient with CRCC with sarcomatoid differentiation who presented a positive response to nivolumab.

## Case presentation

A 41-year-old woman with no medical or family history presented with an incidental right renal tumor. Computed tomography (CT) imaging revealed a 9.5-cm tumor with no evidence of metastatic disease. She underwent right nephrectomy in August 2011. Pathological assessment revealed CRCC with sarcomatoid differentiation, 10.5-cm in maximal diameter and nuclear grade 4 (Fuhrman grade). The pathological stage was T2bN0M0.

Recurrence first occurred in September 2012 with multiple lung masses revealed on CT imaging. In February and August 2013, she underwent metastasectomy twice for the bilateral lung tumors, but recurrence reappeared in February 2014 with multiple lung masses and lung hilar lymph nodes. The pathological result of the lung tumors was also CRCC with sarcomatoid differentiation.

In January 2015, she initiated first-line sunitinib on the 2/1 schedule (37.5 mg once daily for 2 consecutive weeks on treatment followed by 1-week-off), but a drug eruption appeared and the treatment with sunitinib was discontinued. In February 2015, she initiated second-line treatment with pazopanib, 800 mg daily, but the first tumor assessment showed progression of disease. In March 2015, third-line treatment with everolimus was administered, but the disease progressed. In July 2015, fourth-line treatment with sorafenib was administered, but a drug eruption appeared. In September 2015, fifth-line treatment with axtinib was administered, but the disease progressed. In May 2016, sixth-line treatment with temsirolimus was administered, but again, the disease progressed. Her performance status was declining and the symptom of hoarseness from a recurrent nerve paralysis was developing. In July 2016, she decided to receive best supportive care.

**Fig. 1:**
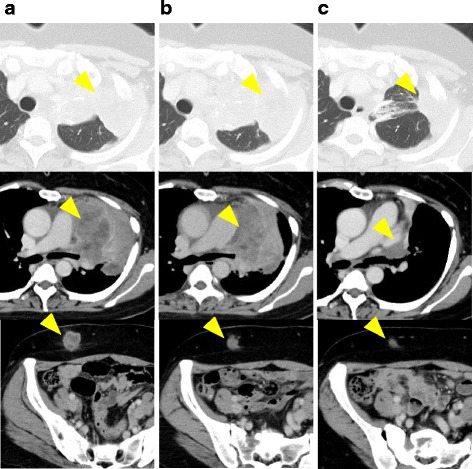
Computed tomography images demonstrate a decrease in the size of lung nodules, lung hilar lymph node metastases, and skin metastases after four and twelve cycles of nivolumab. **a** Before initiating nivolumab therapy. **b** After four cycles of nivolumab. **c** After twelve cycles of nivolumab

In October 2016, nivolumab was approved by pharmaceutical and medical devices agencies in Japan. She initiated seventh-line treatment with nivolumab, 3 mg/kg every 2 weeks, in October 2016. After 4 cycles, a partial response was observed and the symptom of hoarseness was not observed. Significant clinical improvement was noted after 12 cycles (Fig. 1). The treatment has been well-tolerated with no significant side effects thus far, and the patient continues with the treatment of nivolumab at present.

## Discussion

RCC includes multiple histological subtypes. The most common subtype is clear cell RCC (ccRCC) (80.5%), followed by papillary RCC (PRCC) (14.3%), and CRCC (5.2%) [3]. Several reports have suggested that localized non-ccRCC is more likely to have a favorable prognosis than that of ccRCC. Paradoxically, some series have shown that metastatic, non-ccRCC exhibits significantly lower response rates for systemic treatment and poorer median progression-free and overall survival than those with ccRCC [4, 5].

Nivolumab is a fully human IgG4 PD-1 immune checkpoint inhibitor antibody that selectively blocks the interaction between PD-1 and PD-1 ligands 1 (PD-L1) and 2 [1]. In the CheckMate 025 clinical trial, Motzer et al. suggested a superior response rate with nivolumab versus everolimus (25% vs. 5%, respectively) and longer median overall survival (25.0 months vs. 19.6 months, respectively) [1]. However, non-ccRCC patients were excluded in this study, and no prospective trials on the efficacy of immunotherapy in non-ccRCC have been published previously. Little is known about the efficacy of nivolumab in non-ccRCC. Here, we reported, to the best of our knowledge, the second case of a partial response to nivolumab achieved in a CRCC patient.

Only a few case reports have discussed non-ccRCC treated with immune-checkpoint inhibitors. Rouvinov et al. published the first case report on a CRCC patient with sarcomatoid transformation who exhibited a dramatic response to nivolumab as second-line therapy [2]. Geynisman reported the case of a patient with PRCC with sarcomatoid and rhabdoid features who exhibited an excellent response to nivolumab as third-line therapy [6]. Adra et al. reported the case of a patient with unclassified RCC with sarcomatoid features who demonstrated a significant response to nivolumab as second-line therapy [7].

Sarcomatoid differentiation is expressed in 5.1% of ccRCC and 8.2% of CRCC [3]. It has been reported that the presence of sarcomatoid histologic features in RCC is associated with significantly poor prognosis and outcomes for targeted therapies [8, 9]. On the other hand, Joseph et al. reported that PD-L1 positivity in RCC with sarcomatoid differentiation is detected in 89% of patients with these tumors and they may be good candidates for treatment with anti-PD-1/PD-L1 therapy [9]. It has been reported that PD-L1 expression is detected in 23.9% of ccRCC patients [10] and in 10.9% of non-ccRCC patients (5.6% in CRCC, 10% in PRCC, 30% in Xp11.2 translocation RCC, and 20% in collecting duct carcinoma) [4]; PD-L1 expression in RCC with sarcomatoid differentiation is extremely high.

Although PD-L1 expression is predictive of the response to PD-L1/PD-1 inhibitors in patients with lung cancer and melanoma, this association was not established in patients with ccRCC in the CheckMate 025 trial [1, 11, 12]. However, it is unclear whether PD-L1 expression may be a predictive marker for response to immune checkpoint therapy in patients with non-ccRCC. It has been reported that rapidly growing tumors are very likely to respond to anti-PD-1/PD-L1 therapy; although this is opposite to what has been observed previously in the era of molecular targeted therapy [13], there is a possibility that the prognostic factors established thus far may change greatly. The existence of sarcomatoid differentiation may be a predictive marker for the efficiency of nivolumab in non-ccRCC in the era of immuno-oncology. For patients with non-ccRCC with sarcomatoid differentiation that exhibit aggressive characteristics and poor prognosis, nivolumab may be an effective treatment.

## Conclusions

We have reported a case of metastatic CRCC with sarcomatoid differentiation treated with nivolumab as 7th-line therapy with a significant response. Sarcomatoid differentiation may be a predictive marker of the efficiency of nivolumab in patients with non-ccRCC and further investigation is warranted.

## Abbreviations

RCC: Renal cell carcinoma
Ig: Immunoglobulin
CRCC: Chromophobe RCC
Non-ccRCC: Non-clear cell RCC
PD-1: Programmed death 1
CT: Computed tomography
ccRCC: Clear cell RCC
PRCC: Papillary RCC
PD-L1: PD-1 ligands 1
